# Novel 3D local feature descriptor of point clouds based on spatial voxel homogenization for feature matching

**DOI:** 10.1186/s42492-023-00145-4

**Published:** 2023-09-28

**Authors:** Jiong Yang, Jian Zhang, Zhengyang Cai, Dongyang Fang

**Affiliations:** 1 School of Mechanical and Power Engineering, Zhengzhou University, Zhengzhou, Henan 450001 China; 2 School of Management, Hefei University of Technology, Hefei, Anhui 230009 China

**Keywords:** Local feature descriptor, Voxel, Local reference frame, Feature extraction

## Abstract

Obtaining a 3D feature description with high descriptiveness and robustness under complicated nuisances is a significant and challenging task in 3D feature matching. This paper proposes a novel feature description consisting of a stable local reference frame (LRF) and a feature descriptor based on local spatial voxels. First, an improved LRF was designed by incorporating distance weights into Z- and X-axis calculations. Subsequently, based on the LRF and voxel segmentation, a feature descriptor based on voxel homogenization was proposed. Moreover, uniform segmentation of cube voxels was performed, considering the eigenvalues of each voxel and its neighboring voxels, thereby enhancing the stability of the description. The performance of the descriptor was strictly tested and evaluated on three public datasets, which exhibited high descriptiveness, robustness, and superior performance compared with other current methods. Furthermore, the descriptor was applied to a 3D registration trial, and the results demonstrated the reliability of our approach.

## Introduction

3D feature matching through descriptors is an essential yet challenging task in robot navigation and remote sensing [1, 2], medical diagnosis [3], face recognition [4]. This technique is widely deployed in object classification and retrieval [5–8], registration, and reconstruction tasks. In the context of 3D surface matching, the utilization of a high-quality feature descriptor holds utmost importance. This descriptor should possess the capability to effectively extract accurate and consistent information from 3D point clouds, thereby leading to a notable improvement in the overall matching performance. With the development of low-cost 3D scanning devices such as the Photoneo Phixi 3D scanner, Intel Realsence, and Microsoft Kinect, it is now possible to obtain surface point clouds or depth images of objects. However, the original data obtained from ordinary 3D scanners are not flawless and invariably suffer from challenges stemming from varying resolutions of the surface, different levels of noise, inevitable occlusion, clutter, and overlaps in practical industrial applications. However, the large data scale of point clouds also increases the difficulty and complexity of building a stable, efficient, and descriptive descriptor [9, 10]. These practical problems pose major challenges to the development of 3D vision. Therefore, identifying an accurate and robust 3D feature descriptor is a key research concern in this research.

The existing methods for 3D feature descriptors are classified into two main categories: global and local feature descriptors [11–14]. Global feature descriptors extract features from a scene or objects. However, they ignore the shape details of the object and are unable to effectively address occlusion and clutter problems in the scene. Considering this limitation, the target must be segmented from the scene before recognition, making it difficult to satisfy the requirements of complex scene recognition. In contrast, local feature descriptors extract shape features by encoding the geometric and distributional information of neighboring points in histograms or signatures from the confirmed neighboring space. Hence, local feature descriptors are more suitable than global feature descriptors for addressing recognition tasks involving defective objects and complex scenes [15, 16].

Numerous local feature descriptors have been proposed, such as spin image (SI) [17], 3D shape context (3DSC) [18], fast point feature histograms (FPFH) [19], triple orthogonal local depth images (TOLDI) [20], and local voxelized structure (LoVS) [21]. These local feature descriptors can be divided into two categories [16]: feature descriptors without local reference frame (LRF) and feature descriptor with LRF transforms. The feature descriptor method without LRF (e.g., SI, 3DSC, and FPFH) encodes the statistics of local geometric information, such as normal, curvature, and included angle, of surface points in the feature histogram [16, 20]. However, this approach is inadequate for accurately describing the spatial distribution of the local surface and relies only on statistical information. Therefore, this method has limitations in terms of target recognition in complex situations. In contrast, the feature description method with LRF transforms, including rotational projection statistics (RoPS) [16], TOLDI [20], and LoVS [21], solves this problem to some extent. This method establishes the LRF and extracts the local geometric and spatial information in terms of the LRF. Specifically, the LRF is established through the distribution of local neighboring points; therefore, it is entirely determined by the geometric attributes and spatial distribution of the local surface, and it is independent of the world coordinate system. Based on the unique LRF construction, the descriptor invariably performs a rigid transformation and has high descriptiveness because it preserves abundant spatial information of the local surface. Compared to quantitative descriptors without LRF, LRF-based descriptors are superior to descriptors without LRF [13, 22].

Although LRF-based descriptors offer certain advantages, they also have limitations. On the one hand, the feature information outputs of LRF-based descriptors depend strongly on their corresponding LRF [23, 24]. Without an accurate and robust LRF, the output of the feature will be incorrect, and the performance of the descriptors will decrease significantly. In contrast, feature coding also influences the performance of the descriptors. Specifically, the accuracy and stability of the coding method directly determine the descriptiveness and robustness of the descriptors [20].

Considering the aforementioned problems, we propose a local feature descriptor based on voxel homogenization that includes the construction of an accurate and robust LRF and a feature descriptor method with respect to the LRF. First, the weighted covariance matrix was constructed according to the spatial distribution of the neighbors of the keypoint, and the eigenvector corresponding to the minimum eigenvalue of the weighted covariance matrix was selected for determination as the Z-axis. Drawing upon prior research [20], the sum of the projection vectors of all neighboring points was used to determine the X-axis of the LRF. Finally, the Y-axis of the LRF was calculated using the cross-product of the X- and Z-axes. The local surface was subsequently transformed with respect to the LRF, on which all extracted local features rely. For the feature descriptor, the cubic space circumscribed to the sphere neighborhood was assumed and uniformly split into a set of cubic voxels. The label value of each voxel was based on whether it was contained and the number of points in the voxel. If the voxel contained points, its label value was set to 1; otherwise, it was set to 0. To improve robustness and make compensations for LRF deviation in nuisances, the label value of voxels contained in a larger cubic space centered on the voxel with a label value assigned “1” was updated according to the number of points in the center voxel. Finally, the keypoint feature was generated by encoding the label values of all the voxels in terms of the specific spatial index order. To establish the LRF, we chose more appropriate weights to improve descriptiveness and robustness. For feature descriptors, the proposed method ensured not only abundant geometric and distribution information of the local surface via a particular index order but also enhanced the robustness to noise, mesh decimation, and LRF deviation caused by any nuisances through unique label values. To validate the performance of the proposed descriptor, we conducted a series of experiments using three public datasets and compared the results with state-of-the-art datasets. The experimental results demonstrated excellent performance compared to the current methods. The registration results further confirmed the superiority of our descriptor. The main contributions of this study are summarized as follows.

1. An improved LRF construction method is proposed. We set appropriate weights for the determination of the LRF, which can achieve better descriptiveness and robustness in a variety of complicated disturbed environments, such as, noise, varying surface resolution, occlusion, and clutter.
2. A feature descriptor based on voxel label homogenization is proposed, which achieves surpassing performance compared to the existing methods according to the experiment results.

The remainder of this paper is organized as follows: “Related work” section presents a brief literature review of local feature descriptors, including the current LRF construction methods and feature descriptors. “Methods” section introduces the proposed descriptor method. “Results and discussion” section presents the experimental results for three public datasets and a brief evaluation of the proposed method and other state-of-the-art descriptors. Finally, “Conclusions” section concludes this paper.

## Related work

This section briefly introduces the current advanced descriptors, including LRF construction methods and feature descriptor methods. In the proposed method, LRF construction is performed before feature extraction and plays an important role in descriptor approaches. Therefore, we first review the methods used for LRF construction. Feature descriptor reviews, including feature descriptors without LRF and LRF-based, are divided into two categories and described respectively.

### Basic idea of LRF

The LRF is a coordinate system used to compute local feature descriptors to describe the directional and rotational properties of the local region around a keypoint. Before computing the local feature descriptors, it is necessary to determine the LRF for each key point. Once the orientation of the LRF is determined, the local feature descriptors can be computed relative to that of the reference frame.

The choice of LRF is crucial for the performance and robustness of local feature descriptors. An accurately estimated LRF can offer invariance to rotation, scale, and affine transformations, rendering descriptors stable and distinctive across different viewpoints and lighting conditions. The selection and computation of a LRF may vary depending on the specific local feature descriptor. Different algorithms and methods may employ different strategies to estimate and represent the LRF depending on the application requirements and data type.

### Methods of LRF construction

Building a robust, repeatable, and accurate LRF is a fundamental yet challenging task for feature descriptors [16, 22]. Most methods determine an axis using a covariance or scatter matrix generated via a keypoint with its neighbors. Specifically, the Z-axis is usually defined by a normalized eigenvector corresponding to the minimal eigenvalue of the above matrix. Zhong [25] proposed an LRF whose three axes were defined by three eigenvectors obtained by covariance matrix analysis. However, the performance of the LRF is affected by sign ambiguity. Mian et al. [26] used an LRF similar to Zhong’s but addressed the sign ambiguity of the Z-axis. Moreover, Tombari et al. [23] employed a keypoint to replace the centroid of neighboring points to construct a scatter matrix; they augmented the matrix with a distance weight, thereby enhancing its robustness and proposed a principle to disambiguate sign ambiguity. With the appropriate weight used in the scatter matrix and to address sign ambiguity, this method achieves strong robustness to noise, while offering sensitivity to varying mesh resolutions [16, 20]. Petrelli and Di Stefano [15] studied the repeatability of the LRF on occlusion and local surface boundaries, using the normal of the points at the boundary to determine the X-axis and robustness to the surface boundary. Furthermore, Guo et al. [16] proposed a novel method using the weighted triangles of the local surface to build a scatter matrix and sign disambiguation to enhance the performance of the LRF. This method exhibits high accuracy and robustness to noise; however, it is a protracted process owing to the computation of the triangle construction. Motivated by the literature [15], Yang et al. [20] selected a small subset of neighboring points to generate a covariance matrix, and they introduced a novel method for calculating the X-axis using the sum of the weighted projected vectors. This method exhibits low ambiguity of the X-axis and offers robustness to occlusion and boundaries owing to the choice of a subset of neighboring points. However, this method suffers from low compactness in terms of the feature description [27]. Ao et al. [27] adopted a Gaussian function weight and distance weight in a covariance matrix to enhance the performance of the LRF. Although their approach allows for high stability, the calculation complexity involved in this method rises significantly.

The aforementioned methods offer different advantages and are effective in solving the problems of ambiguity, noise, occlusion, and varying surface resolution. However, these methods suffer from one or more limitations, and it is difficult to achieve excellent overall performance.

### Methods of descriptor

Numerous attempts have been made to leverage local feature descriptors in research fields, which are mainly classified into two categories, as mentioned in Introduction section. We introduce these two categories successively, as follows.

#### Descriptors without LRF

Johnson and Hebert [17] proposed the SI descriptor, which was one of the earliest 3D local feature descriptors. This descriptor defines the normal of the local surface as a reference axis and spins a 2D plane around the axis, which is then divided into several bins. The number of points falling in each bin is summed as the gray value of a 2D image. Although SI is widely cited and time-efficient approach, it is sensitive to mesh resolution and has limited descriptiveness [16, 20, 27]. Chen and Bhanu [28] proposed a local surface patch (LSP) descriptor, which constructs LSPs and encodes the shape index and normal deviations of neighbors into a feature histogram. The LSP descriptor is efficient but still vulnerable to noise and varying mesh decimation [16, 22]. Rusu et al. [29] presented a point feature histograms (PFH) by using the geometric angle relationship of point pairs between keypoints and neighboring points. The PFH has a higher descriptiveness than the SI, but its time efficiency is relatively lower. To solve this problem, Rusu et al. [19] proposed the FPFH descriptor, which calculates the simplified point feature histograms (SPFH) of the k-neighboring points of the keypoint and encodes all weighted SPFH into the FPFH. Moreover, it is a time efficient and distinctive approach [16, 20, 22]. Flint et al. [30] introduced the THRIFT descriptor, which calculates the deviation angles between the keypoint and the normals of neighboring points to generate a 1D normalized histogram. THRIFT also lacks robustness against noise [22]. Frome et al. [18] proposed a 3DSC descriptor that divides the spherical neighboring space according to a reference axis and then counts the number of weighted points that fall into each bin as the output feature. However, it is not rotationally invariant, and depends only on the reference axis.

Descriptors without LRF extract and characterize features using geometric relative invariants, such as the angle between points and normal and the distance between points, to generate feature histograms. Compared with LRF-based descriptors, their performance is limited owing to the lack of spatial information.

#### Descriptors with LRF

For descriptors with LRF, Zhong [25] proposed an LRF via three eigenvectors obtained through covariance matrix analysis and further proposed the intrinsic shape signatures (ISS) descriptor to characterize the local features by accumulating the number of weighted points in spherical neighborhood surface grids. The performance of the ISS descriptor was not remarkable owing to the sign ambiguity of the LRF. Tombari et al. [31] developed a 3DSC descriptor for the unique shape context (USC) descriptor by employing a disambiguated LRF and dividing the neighborhood space into grids along the azimuth, elevation, and radial directions. With an accurate and stable LRF, the USC exhibits outstanding robustness to noise and is sensitive to varying mesh decimations [22]. Subsequently, Tombari et al. extended the USC descriptor to the signature of histograms of orientations (SHOT) via deploying deviation angles between the normal of a keypoint and its neighboring points [16, 20, 22]. Although it is robust to noise and time efficiency, the SHOT descriptor is limited by mesh resolution variation. Guo et al. [16] proposed a RoPS descriptor for local characterization and object recognition. The RoPS descriptor extracts feature information by calculating the density of neighboring points and the Shannon entropy with respect to numerous rotations of the local surface around each axis. Despite its superior descriptiveness, the RoPS descriptor is limited by extremely time-consuming and nonuniform points [13, 20]. Similar to the view-based method of the ROPS descriptor, Guo et al. [14] advanced the SI descriptor and proposed the tri-spin-image (TriSI) feature descriptor. The TriSI descriptor was generated by merging three spin-image signatures that were calculated based on the coordinate axes of the LRF. TriSI is more robust to occlusion and clutter than RoPS; however, this approach is constrained by its high time consumption [20]. Yang et al. [20] introduced the TOLDI descriptor, which was created by integrating the feature information of three normalized local depth images acquired from projection planes perpendicular to each axis of the LRF. With an excellent LRF and a reasonable encoding method, the TOLDI descriptor achieves extraordinary performance in terms of descriptiveness and robustness; however, it suffers from low compactness of the feature description [27, 32]. Tang et al. [33] proposed the signature of geometric centroids (SGC) descriptor, which serves as a novel approach to spatial cubic voxelization for feature generation. The SGC descriptor voxelizes the neighboring space of the local surface into cubic voxels, counts the number of points in each voxel as the voxel label, and encodes label values to the feature vector according to a specific principle. The strategy of cubic voxelized neighboring space is convenient for uniform spatial segmentation to extract features, which are reflected on the voxel label that not only reserves the spatial distribution of points but also recodes the geometric information of the local surface. Quan et al. [21] extended the SGC descriptor to a LoVS descriptor. The LoVS descriptor splits the neighborhood space into a set of cubic voxels, similar to the voxelized strategy of the SGC. The label of the voxel is determined based on whether it contains points, and subsequently, all voxel labels are encoded to the feature vector. The LoVS descriptor is suitable for low-quality point clouds and is an efficient technique; however, it is also limited by its relatively low compactness. Zhao et al. [32] proposed the statistic of deviation angles on subdivided space (SDASS) descriptor, which extracts the distribution information of neighboring points and encodes feature histograms based on a novel local reference axis and local minimum axis. The SDASS descriptor achieves high robustness to noise and varying surface resolutions. Sun et al. [34] introduced a weighting function to improve the stability of the description method according to the theory of feature space optimization and to improve the compactness of feature information by varying the size of the support radius. Bai et al. [35] proposed a joint framework to extract description features while extracting keypoints, which improved the computational efficiency of the feature description and extraction process. Ao et al. [36] used a deep learning method to extract the local features of a point cloud and generated a cylindrical voxel based on a spherical neighborhood. They performed convolution calculations based on this approach, which improved the calculation speed of feature extraction.

Although the aforementioned descriptors have various degrees of descriptiveness and robustness to noise, different mesh decimations, and occlusions, none can effectively deal with multiple challenges.

## Methods

This section introduces the novel feature-description method in detail. Considering that this description method contains two parts: an LRF construction method and a feature representation of the SVH, this section introduces these two parts in turn. First, we describe the construction method of the modified LRF in detail. It is based on the normal of the keypoint and sum of the weighted projection vectors. Subsequently, we present the feature representation by encoding homogenized spatial voxel labels based on the proposed LRF. Finally, the parameters of the descriptor were selected via quantitative testing and analyses.

### LRF construction

**Fig. 1:**
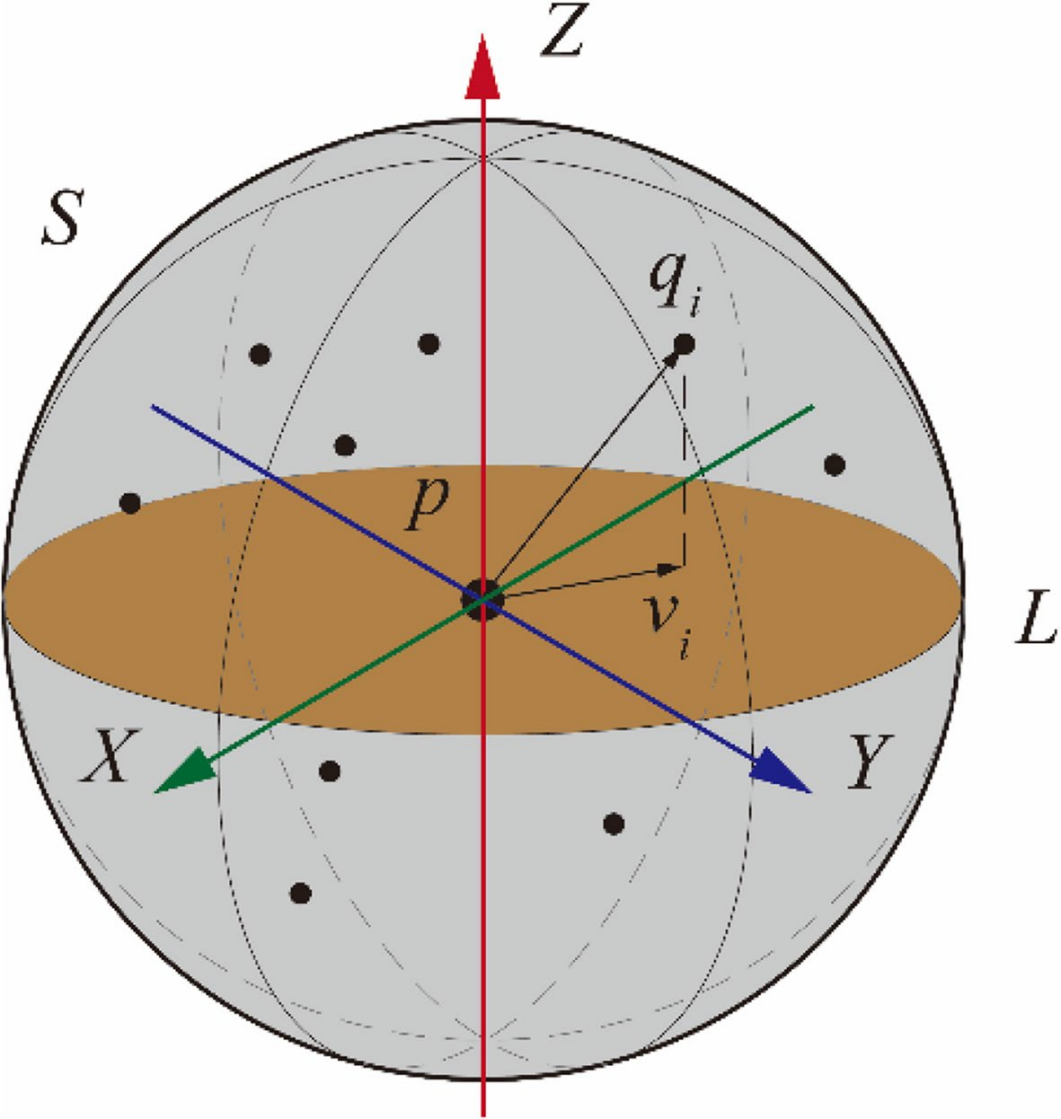
Sketch map of the method of LRF construction. The blue point and black points denote the keypoint *p* and neighboring points *q*_*i*_ within the neighborhood with support radius *r*, respectively. The orange plane denotes the projective plane perpendicular to Z-axis. The blue, red, and green arrows denote the Z-axis of LRF, vectors from *p* to *q*_*i*_, and the vector projecting to plane L

First, we introduce the construction method for the modified LRF in detail, as shown in Fig. 1. The LRF at keypoint *p* is composed of three vector coordinate axes that are orthogonal to each other in space:

1 $${\varvec{F}}=\left\{{\varvec{F}}.{{\varvec{x}}}_{{\varvec{p}}},{\varvec{F}}.{{\varvec{y}}}_{{\varvec{p}}},{\varvec{F}}.{{\varvec{z}}}_{{\varvec{p}}}\right\}$$

where **F**, **F.x**_**p**_, **F.y**_**p,**_ and **F.z**_**p**_ represent the LRF and its X-, Y-, and Z-axes, respectively. Considering that the Y-axis of the LRF can be obtained by the cross-product of the Z- and X-axes, determining the stable and accurate Z- and X-axes is the core function in LRF construction.

Specific illustration of our LRF construction method is as follows:

Given a keypoint *p* and a spherical neighboring space with radius *r* centered on *p*, the neighboring points *q*_*i*_ of *p* within neighborhood S constitute a point set D = {*q*_*1*_*, q*_*2*_*, …, q*_*n*_}. The centroid *c* of D is defined as

2 $$c=\frac{1}{n}{\sum }_{i=1}^{n}{q}_{i}$$

The covariance matrix **M** is constructed using coordinates *c* and *q*_*i*_ to describe the distribution of *q*_*i*_ around *c*, as follows:

3 $${\varvec{M}}={\sum }_{i=1}^{n}{G}_{i}({q}_{i}-c{)}^{T}\left({q}_{i}-c\right)$$

4 $${G}_{i}=r-\left|\left|{\varvec{p}}{{\varvec{q}}}_{{\varvec{i}}}\right|\right|$$

where *G*_*i*_ is the weight of the covariance matrix **M**, ||•|| denotes the L2 norm, and **pq**_**i**_ is the vector from keypoint *p* to neighboring point *q*_*i*_, whose L2 norm value represents the Euclidean distance from *p* to *q*_*i*_ and is a significant relative invariant for rigid rotation and translation in the spherical neighborhood. To improve the robustness of the LRF to varying mesh resolutions and noise, we employed *G*_*i*_, which implies a larger distance from *p* to *q*_*i*_ and the few influences it exerts on the LRF [23]. Thus, adding this weight can effectively improve the validity and distinguishability of the covariance matrix analysis results. The stability of the LRF increased with this weight. The detailed results are presented in Theoretical analysis of methods section.

After the covariance analysis, three eigenvalues {e_1_, e_2_, e_3_} and their corresponding eigenvectors {v_1_, v_2_, v_3_} were obtained. The eigenvector corresponding to the minimum eigenvalue is used to define the direction of the Z-axis on *p*. Note that there is a difference between the normal direction, which is calculated by the covariance matrix without weight, and the Z-axis owing to the employment of weight *G*_*i*_ for **M**. However, the direction of the Z-axis of *p* is always affected by sign ambiguity, which is related to the repeatable performance of the LRF. To solve this problem, we define the disambiguated Z-axis of *p* as

5 $${\varvec{F}}.{{\varvec{z}}}_{{\varvec{p}}}=\left\{\begin{array}{c}{{\varvec{z}}}_{{\varvec{p}}},if{\sum }_{i=1}^{n}{{\varvec{z}}}_{{\varvec{p}}}\cdot {\varvec{p}}{{\varvec{q}}}_{{\varvec{i}}}\cdot {G}_{i}\ge 0\\ -{{\varvec{z}}}_{{\varvec{p}}},otherwise\end{array}\right.$$

where **z**_**p**_ represents the direction of the Z-axis.

To determine the X-axis, we referred to the method described by Yang et al. [20]. Based on the tangent plane L of *p* determined with respect to **F.z**_**p**_, the projection vector **v**_**i**_ of **pq**_**i**_ is obtained by

6 $${{\varvec{v}}}_{{\varvec{i}}}={\varvec{p}}{{\varvec{q}}}_{{\varvec{i}}}-{\varvec{F}}.{{\varvec{z}}}_{{\varvec{p}}}\cdot \left({\varvec{F}}.{{\varvec{z}}}_{{\varvec{p}}}\cdot {\varvec{p}}{{\varvec{q}}}_{{\varvec{i}}}\right)$$

The weight selected available can be considered from five aspects:

- *v*_*i*_ is the Z-axis height of the corresponding vector **pq**_**i**_, i.e., ${\mathbf{pq}}_{{\mathbf{i}}} \cdot {\mathbf{F}}{\mathbf{.z}}_{{\mathbf{p}}}$;
- For the spatial distribution of point *q*_*i*_, the surface distribution of the point cloud is similar to a Gaussian distribution under a dense surface resolution. For Gaussian function $G(||{\mathbf{pq}}_{{\mathbf{i}}} ||) = \frac{1}{{\sqrt {2\pi \sigma^{2} } }}e^{{ - \frac{{(||{\mathbf{pq}}_{{\mathbf{i}}} || - \mu )^{2} }}{{2\sigma^{2} }}}}$, where *μ* and *σ* mean and variance of ||**pq**_**i**_||, respectively. Although adding a Gaussian distribution weight can improve the stability of the LRF [16], the weight is rendered invalid owing to the lack of sufficient sample points under a low surface resolution.
- Distribution of projection points in the projection plane: it is difficult for the distribution of points in the projection plane to converge to a certain distribution under low surface resolution; therefore, it is not considered.
- The module information of the **pq**_**i**_: to improve the robustness of the LRF to occlusion and stacking, it is formulated as $r - ||{\mathbf{pq}}_{{\mathbf{i}}} ||$. Thus, the X-axis is defined as the sum of all weighted projection vectors, as follows:
  7 $${\varvec{F}}.{{\varvec{x}}}_{{\varvec{p}}}=\frac{{\sum }_{i=1}^{n}{w}_{i1}{w}_{i2}{{\varvec{v}}}_{{\varvec{i}}}}{{\sum }_{i=1}^{n}|{w}_{i1}{w}_{i2}{{\varvec{v}}}_{{\varvec{i}}}|}$$
  8 $${w}_{i1}=(r-\left|{\varvec{p}}{{\varvec{q}}}_{{\varvec{i}}}\right|{)}^\frac{1}{2}$$
  9 $${w}_{i2}=({\varvec{p}}{{\varvec{q}}}_{{\varvec{i}}}\cdot {\varvec{F}}.{{\varvec{z}}}_{{\varvec{p}}}{)}^{2}$$

where *w*_*i1*_ is related to *r* and **pq**_**i**_, which enhance the robustness to clutter, occlusion, and surface boundaries. The second weight *w*_*i2*_ represents the projection length of **pq**_**i**_ along the Z-axis, which improves the distinguishability of the projection vector [20].

Finally, the Y-axis of the LRF was determined by the cross-product of the Z- and X-axes. It is possible to generate a local feature descriptor using this unique and highly descriptive LRF.

10 $${\varvec{F}}.{{\varvec{y}}}_{{\varvec{p}}}={\varvec{F}}.{{\varvec{x}}}_{{\varvec{p}}}\times {\varvec{F}}.{{\varvec{z}}}_{{\varvec{p}}}$$

### Method of feature descriptor

**Fig. 2:**
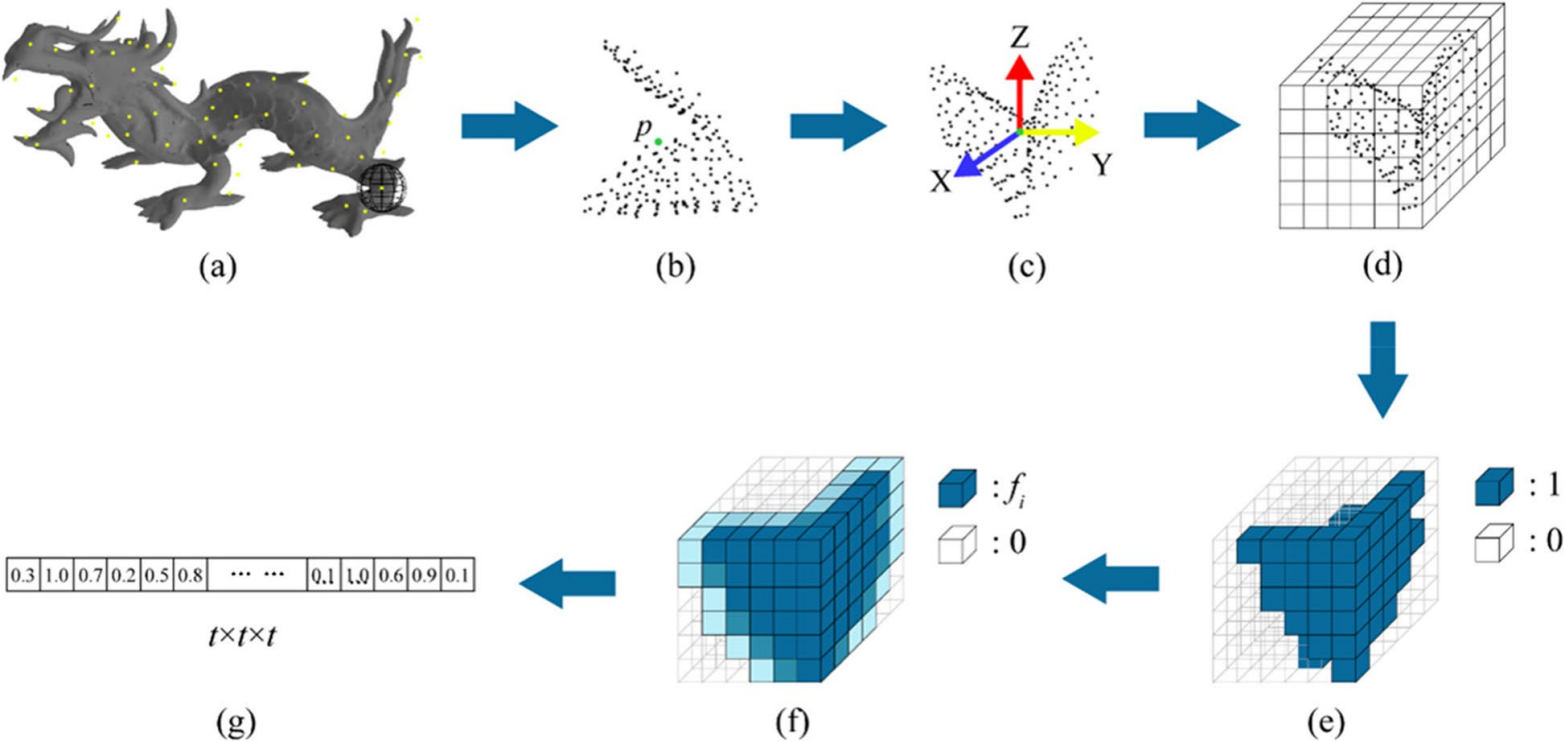
Illustration of descriptor principle. (**a**) Original 3D object, where yellow points represent the keypoints obtained from the surface of the object; (**b**) The local surface within neighboring space with radius *r* and centered on keypoint *p*; (**c**) Construction of LRF on keypoint *p* and transformed local points with respect to the LRF, where blue, yellow, and red arrows represent X-, Y-, and Z-axis, respectively; (**d**) Split space into a set of cubic voxels; (**e**) Set label for each voxel, where blue voxels and transparent voxels represent label value is 1 and others is 0, respectively; (**f**) Update the label value of each voxel, where the color depth of the cube represents the size of voxel label value; (**g**) Output feature of keypoint *p* by merging label value of each voxel according to particular index coding

The general descriptor process is illustrated in Fig. 2. After LRF construction, we transform the local surface neighboring points of keypoint *p* with respect to LRF F for feature description and extraction, as shown in Fig. 2(c). We construct the transform matrix ${\mathbf{T}} = {\mathbf{F}}$ using the LRF, **F** as the rotation matrix and translation matrix **t** to convert the neighboring points of the keypoint into a new coordinate system, as follows:

11 $${\text{D}}_{{\text{T}}} = {\mathbf{T}} \cdot ({\text{D}} - p)$$

where D_T_ = {*q*′_1_, *q*′_2_, …, *q*′_*n*_} denotes the normalized local surface transformed with respect to **T**. Then, we consider the circumscribed cubic space of the sphere neighborhood of the keypoint as the new neighborhood space. On the one hand, compared with a spherical neighborhood or other geometric neighborhoods, the cubic neighborhood space has several obvious advantages. In particular, the cubic neighborhood facilitates uniform voxelization. On the other hand, uniform and equal spatial voxelization better preserves the spatial distribution of points and geometrical local shapes and addresses information asymmetry problems such as redundancy or sparseness of spatial information caused by uneven voxelization in feature representation and extraction. We split the cubic neighborhood space *g*-1 times uniformly into N = *g* × *g* × *g* spatial voxels along the edge direction of the cube, and the length of each voxel *l* was $\frac{2r}{g}$.

To facilitate the expression of spatial features, we referred to the voxel index coding method of Quan et al. to create an index *I* for each voxel *v* [21]:

12 $$I_{{v_{i} }} = \left\lfloor {\frac{{{\mathbf{p^{\prime}q^{\prime}}}_{{\mathbf{i}}} .z + r}}{l}} \right\rfloor \cdot g^{2} + \left\lfloor {\frac{{{\mathbf{p^{\prime}q^{\prime}}}_{{\mathbf{i}}} .y + r}}{l}} \right\rfloor \cdot g^{1} + \left\lfloor {\frac{{{\mathbf{p^{\prime}q^{\prime}}}_{{\mathbf{i}}} .x + r}}{l}} \right\rfloor \cdot g^{0}$$

where ${\mathbf{p^{\prime}q^{\prime}}}_{{\mathbf{i}}}$ represents the transformed **pq**_**i**_ vector.

We counted the point set Q_i_ of each voxel and assigned all voxels the label *l*_*i*1_ according to whether Q_i_ is empty, based on the following expression:

13 $$l_{i1} = \left\{ \begin{gathered} 1 \, ,{\text{ if Q}}_{{\text{i}}} { > 0} \hfill \\ {\text{0 , if Q}}_{{\text{i}}} { = 0} \hfill \\ \end{gathered} \right\}$$

**Fig. 3:**
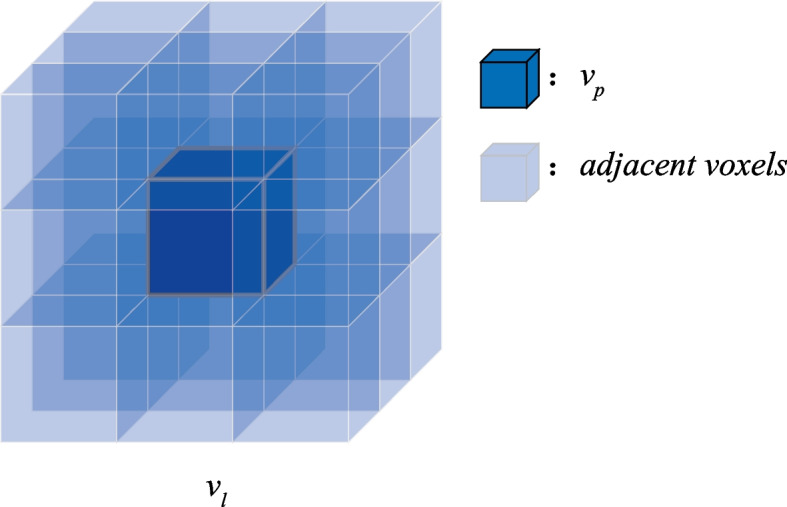
The adjacency relationship of voxels when *w* is 3. *v*_*p*_ is the center voxel whose voxel label value is “1” in the large voxel* v*_*l*_. The adjacent voxels of *v*_*p*_ are represented in light blue

**Fig. 4:**
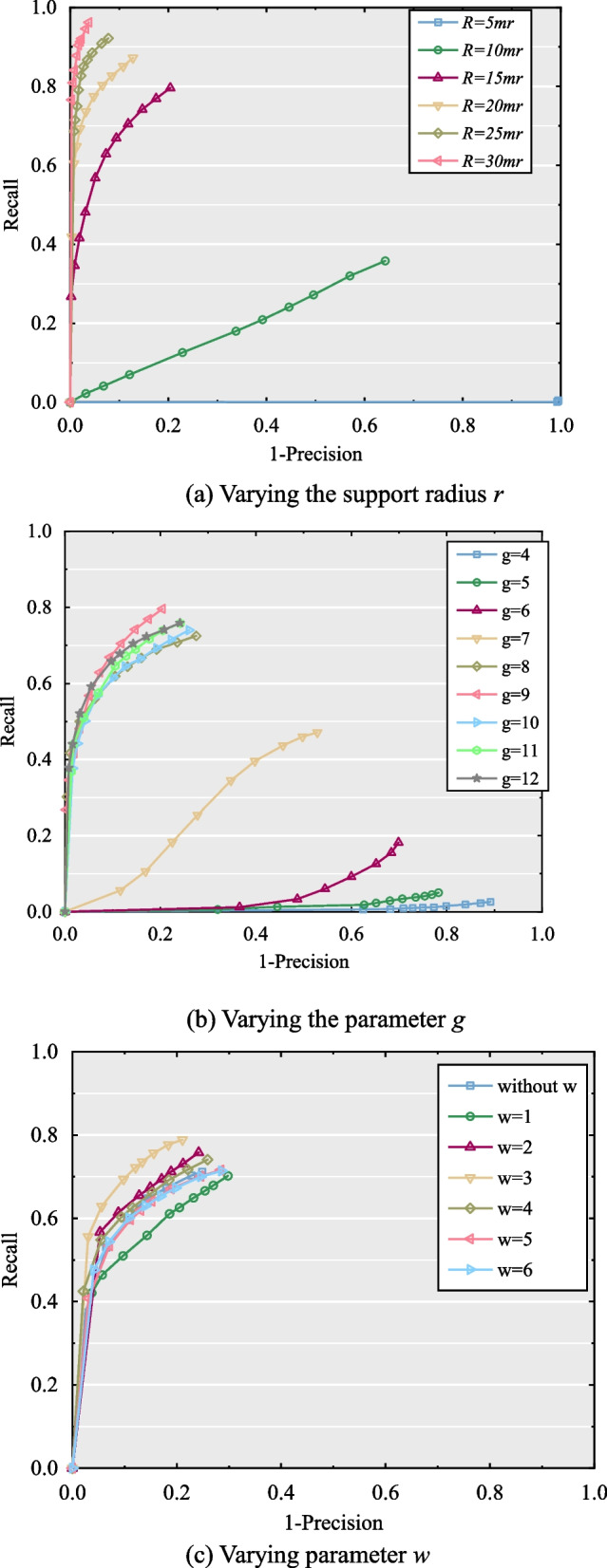
The parameter settings of the proposed descriptor. (**a**) Varying the support radius *r;* (**b**) Varying the parameter *g;* (**c**) Varying parameter *w*

As shown in Figs. 3 and 4, according to the adjacency relationship, each voxel comprises (*w*^3^-1) adjacent voxels, where *w* is the number of adjacent voxels of a large voxel *v*_*l*_ centered on *v*_*p*_, which is along the edge direction of *v*_*p*_. After calculating label *l*_*i*1_, we assigned the adjacent voxels of *v*_*p*_ to a new label *l*_*i*2_:

14 $$l_{i2} = \frac{{n_{i} }}{{w^{3} }}$$

15 $$l_{i3} = \sum\limits_{i = 1}^{{w^{3} }} {l_{i2} }$$

where the* n*_*i*_ is the number of points in *v*_*p*_. *v*_*l*_ contained *w* × *w* × *w* voxels. Subsequently, the label value *l*_*i*2_ of every voxel is updated, and *l*_*i*3_ is calculated by summing the label values of the adjacent voxels for every voxel. The robustness of the descriptor to noise, varying distribution of the surface, and deviation of the LRF are improved in this step.

The feature value of each voxel is composed of its *l*_*i*1_ and *l*_*i3*_:

15 $$f_{i} = l_{i1} + l_{i3}$$

Finally, all the labels of each voxel were recoded into features of *p* according to index *I*.

16 $$f_{p} = [f_{1} ,f_{2} ,...,f_{N} ]$$

We encoded the three-dimensional space voxels and their labels into a one-dimensional feature number string. Although there is an inevitable loss of information in the process (Eq. 16), this method guarantees computational efficiency. Compared to the binarized one-dimensional feature descriptor, our method has a slightly lower operating efficiency; however, it obtains stronger robustness to noise, varying surface distribution, and occlusion. In contrast, the spatial voxelization method reduces the interference of the calculation error of the LRF on feature extraction and description to a certain extent; therefore, this method offers a certain level of stability.

### Theoretical analysis of methods

We performed a preliminary analysis of the theoretical level of algorithm performance, and the results are presented herein.

#### Descriptive ability

The description method fully considers the relative invariants of all neighboring points of the keypoint in the LRF construction and the appropriate weight. Specifically, the distance from neighboring points to the edge of the neighborhood was used as the weight to enhance the descriptiveness and stability of the Z-axis of the LRF. In addition, appropriate geometric attribute weights were used to determine the X-axis to improve the discrimination and uniqueness of the X-axis. The descriptor guarantees the consistency of each unit voxel in the spatial volume by uniformly splitting the spatial space, and ensures the equivalence of the spatial information of each spatial voxel. Based on these advantages, each voxel was assigned a label according to the spatial distribution of the surface points, and all voxel labels were encoded into one-dimensional features. The encoding sequence reflects the relative position distribution of spatial voxels, which is unique and descriptive. Therefore, it exhibits excellent space description performance.

#### Stability

This method leverages different weights to improve the adaptability and stability of the LRF to noise, local boundaries, and occlusions in various scenarios. To a certain extent, the spatial voxels constructed by the descriptor were similar to those constructed by image filtering in 2D image processing. Specifically, noise and truth points have a certain probability of falling into the same voxel during feature extraction. Therefore, noise has little impact on the overall feature extraction. Moreover, feature updating is a smoothing process that reduces the interference of noise. In general, this method can eliminate or reduce the influence of noise within a certain range of feature descriptors.

#### Invariance to rigid transformations

The LRF construction method builds a covariance matrix using the distance from each neighboring point of the keypoint to the centroid and center. These distances are relatively invariant for rigid transformations. Therefore, the LRF is entirely defined by the geometric attributes and spatial distribution of neighboring points and is independent of the world coordinate system. Based on this unique LRF construction, the descriptor inherits invariance to a rigid transformation.

#### Computational efficiency

The time efficiency of the descriptor is primarily influenced by the size of the matrix in the LRF construction and the number of voxels in the feature computation. Compared with other algorithms, the time efficiency of the proposed algorithm is at an intermediate level.

### Parameters of descriptor

A few parameters and variables affect descriptor performance. Specifically, the support radius *r,* parameter *w* with respect to the feature label, and parameter *g* which is related to the voxel number N, the weights used to determine Z- and X-axes in constructing the LRF, have a significant impact on the performance of the descriptor. Note that the support radius *r* affects the performance of both the LRF and the descriptor [20, 32]. With large values of *r*, the computational efficiency of the LRF construction and descriptor will be high because of the massive number of neighboring points, as well as being more sensitive to occlusion and boundary regions. However, the information density will be lower, leading to reduced descriptiveness for small values of *r*. Therefore, it is necessary to select a suitable size value of *r* to guarantee the performance of the descriptor. Furthermore, the parameter *w* is critical to the feature value. Based on a larger or smaller value of *w*, the feature label *l*_*i*2_ is minimized and loses its effect. Moreover, parameter *g* determines the number of voxels related to the information density of each voxel. On the one hand, a large value of *g* enhances descriptiveness owing to the high accuracy of spatial information, whereas it is time-consuming and sensitive to noisy or varying densities of the local surface [21]. However, for a small value of radius *r*, the descriptor will not be sufficient for extracting sufficient feature information. Additionally, the weight used in LRF construction affects the performance of the LRF. In this context, a reasonable weight helps improve the robustness and descriptiveness of the descriptor.

To set the appropriate parameters of the proposed descriptor, the test was conducted on the scene with 1/4 mesh decimation and 0.3 *mr* (*mr* hereinafter, mesh resolution) Gaussian noise in the B3R dataset. To evaluate the performance of the descriptor quantitatively under various parameter settings, we employed the recall vs 1-precision curve (RPC; see the detailed introduction in Results and discussion section). The support radius *r* and parameter *g* were set varying from 5 to 30 *mr* and 4 to 12, respectively. For the weight test, we used the error curve to evaluate the influence of different weights and confirmed that the weight we used was reasonable.

**Fig. 5:**
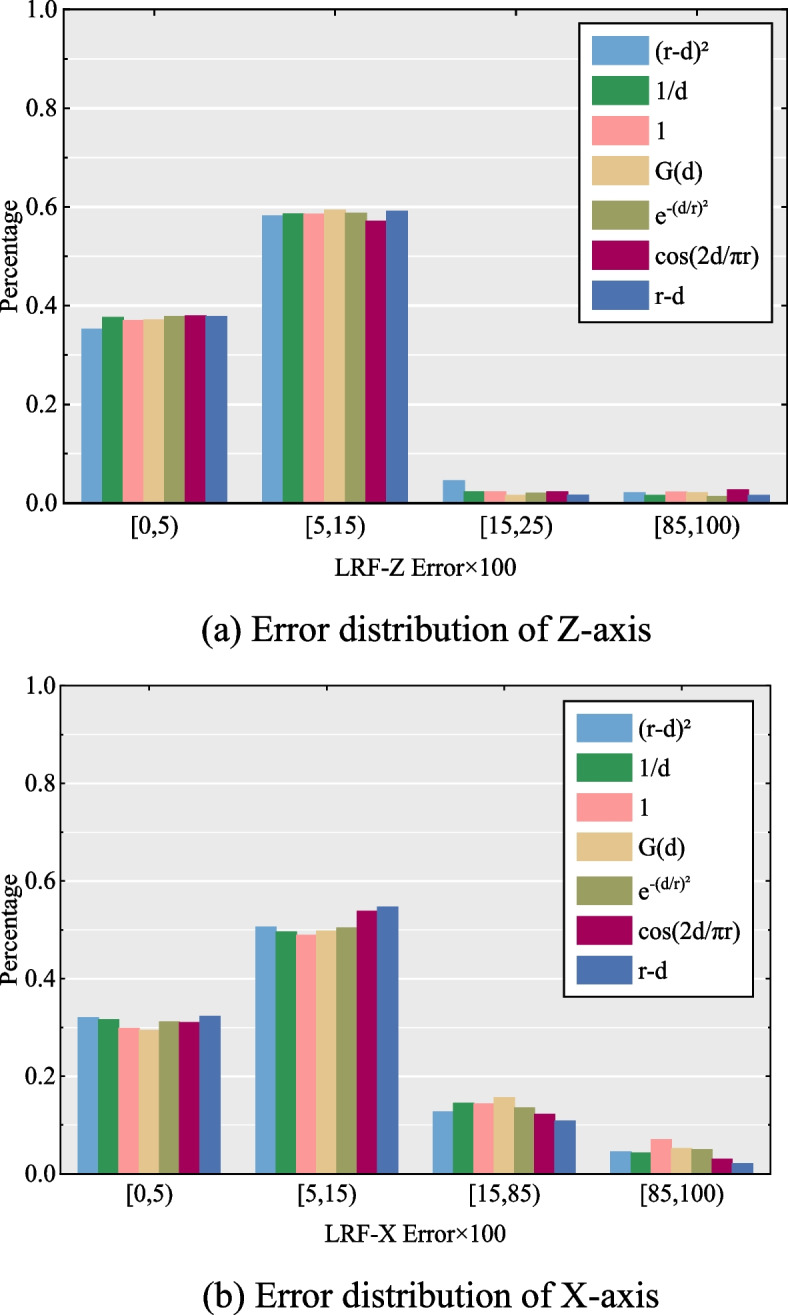
The setting test of weights in the covariance matrix of LRF construction of the proposed descriptor. (**a**) Error distribution of Z-axis; (**b**) Error distribution of X-axis

The experimental results for the parameters and weights are shown in Figs. 3, 4 and 5, respectively. The parameter test results reveal that the performance improves gradually as the support radius *r* increases from 10 to 30 *mr*, except for 5 *mr* due to less information in a small neighboring space. However, with a larger *r*, the performance of the descriptor increases and the computational efficiency decreases. Therefore, it is reasonable to set the support radius *r* to 20 *mr*. For parameter *w*, the performance increases when improves the value of *w* progressively, whereas it decreases when *w* is larger than three. Consequently, we set *w* to 3 in this study. For parameter *g*, it is evident that the performance increases as *g* increases from 4 to 9, whereas it decreases when *g* is greater than 9. The performance of the descriptor decreases because of its sensitivity to noise and mesh decimation with *g* larger than 9. Thus, parameter g was set to 9 in this study.

**Fig. 6:**
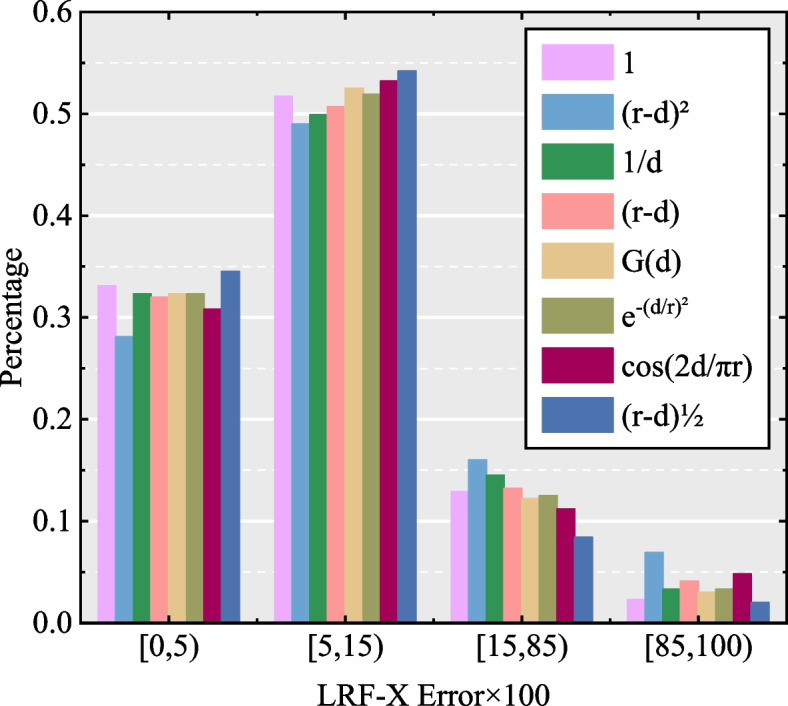
The setting test of weights which applied to determine the X-axis of LRF construction of the proposed descriptor

For the weight employed in the covariance matrix of the LRF construction, we tested five different weights: (*r*–*d*_*i*_)^2^ (where *d*_*i*_ =||*q*_*i*_ – *p*||, the following is the same), 1/*d*_*i*_, G(*d*_*i*_) (where $G(d_{i} ) = \frac{1}{{\sqrt {2\pi \sigma^{2} } }}e^{{ - \frac{{(d_{i} - \mu )^{2} }}{{2\sigma^{2} }}}}$ is a Gaussian function), $e^{{ - (d_{i} /r)^{2} }}$, cos(2*d*_*i*_/π*r*), (*r*–*d*_*i*_), and 1, which means no weight. In the aforementioned weights, (*r*–*d*_*i*_)^2^, (*r*–*d*_*i*_), 1/*d*_*i*_, and $e^{{ - (d_{i} /r)^{2} }}$ indicate that distant neighboring points have little influence, whereas (*r*–*d*_*i*_)^2^ and (*r*–*d*_*i*_) have been widely adopted in the literature [18, 20, 23, 27]. G(*d*_*i*_) was used in ref. [27]. The distribution curve of the weight cos(2*d*_*i*_/π*r*) is similar to a Gaussian function. To guarantee the validity of the results, we altered the weight used in the covariance matrix and kept the other variables unchanged. The weight test results indicate that these weights exhibit similar performance, whereas the (*r*–*d*_*i*_) weight exerts a superior influence over X-axis determination. Regarding the weights, *w*_*i*1_ was applied to determine the X-axis of the LRF construction, and the error of the X-axis of the LRFs was tested by assigning seven different weights, including the aforementioned weights, and altering the final weight 1 to (*r*–*d*_*i*_)^1/2^. Similarly, we maintain the variable *w*_*i*2_ during testing as this variable offers high descriptiveness. The experimental results are presented in Fig. 6. Consequently, the (*r*–*d*_*i*_)^1/2^ weight realizes remarkable performance in determining the X-axis compared with the other weights. According to these results, the weight in the covariance matrix was set to (*r*–*d*_*i*_), and *w*_*i*1_ was set to (*r*–*d*_*i*_)^1/2^.

## Results and discussion

In this section, the proposed LRF and descriptor are tested on three public standard datasets: the Bologna dataset [23, 37, 38], Bologna 3D retrieval dataset [39, 40], and University of Western Australia UWA 3D object recognition (U3OR) dataset [26, 41]. To provide convincing evaluation results, the proposed descriptor was compared with several advanced current methods under different nuisances, including Gaussian noise, varying mesh decimation, and occlusion. All experiments were implemented on a computer with a 2.9 GHz CPU (Intel Core i5-9400F) and 8 GB RAM.

### Experimental setup

#### Datasets

The Bologna dataset for descriptor matching comprised six models and 45 scenes. The models (i.e., “Bunny,” “Armadillo,” “Asia Dragon,” “Happy Buddha,” “Dragon,” and “Thai Statue”) correspond to the Stanford 3D Scanning Repository [42], and the scenes contain a subset of models augmented with rotation and translation. In addition, the datasets also offer configuration files that define the number of models and the names of scenes, along with ground-truth files that define the rotation and translation applied to each model in that scene.

The B3R dataset, consisting of six models and 18 scenes, was established for retrieval. The models were also obtained from the Stanford 3D Scanning Repository, and the scenes are created by the rotated models with three standard Gaussian noises of 0.1, 0.3*,* and 0.5 *mr*. This dataset offers configuration and ground-truth matrix information.

**Fig. 7:**
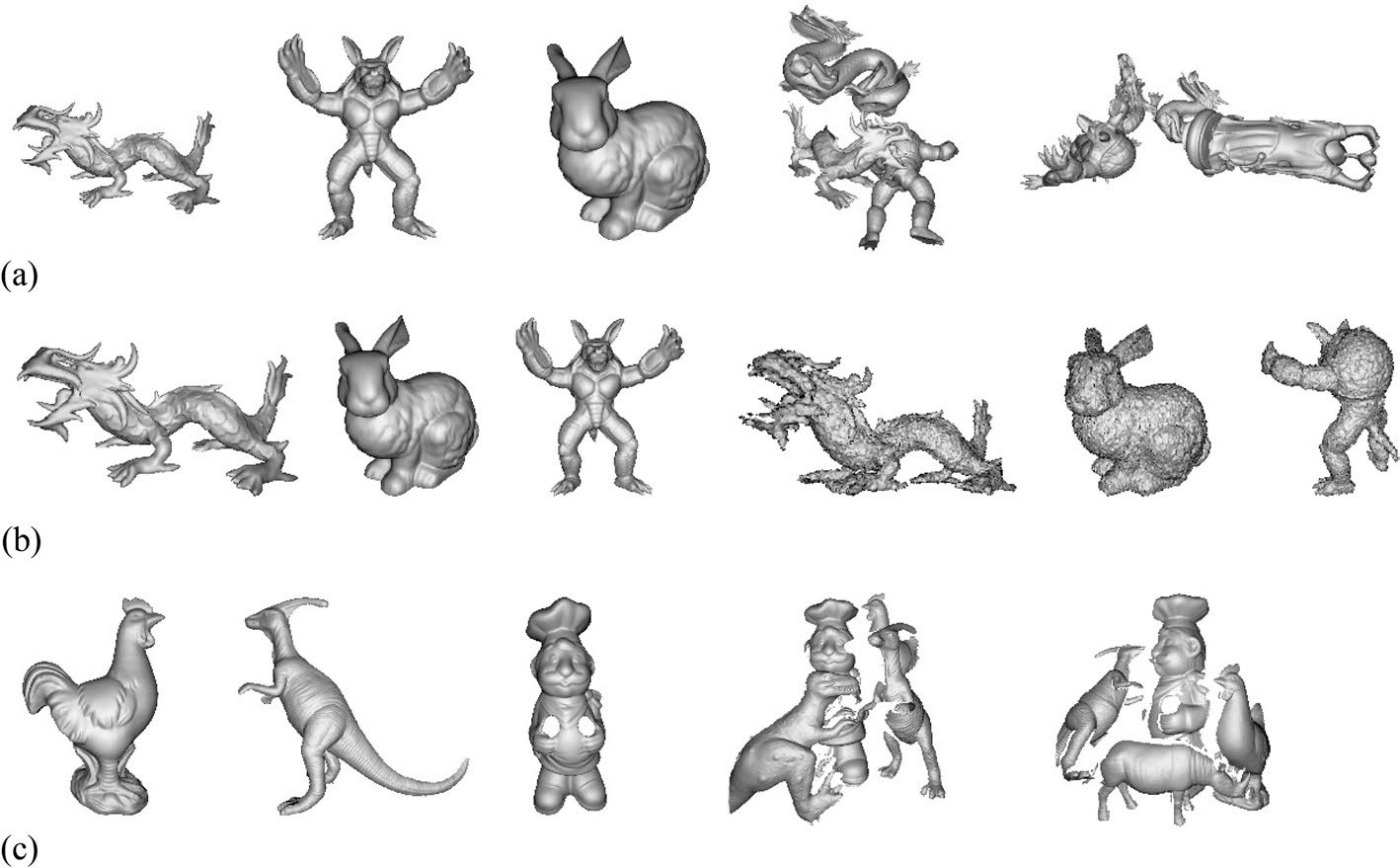
From left to right: three models and three scenes which viewed in mesh representation of (**a**) The Bologna dataset, (**b**) The B3R dataset, and (**c**) The U3OR dataset

The U3OR dataset contains 5 models (i.e., “Chicken,” “T-Rex,” “Parasaurolophus,” “Rhino” and “Chef”) and 50 scenes. The scenes were scanned with several models played randomly using a Minolta Vivid 910 scanner. Note that the different levels of occlusion and clutter of each scene are significant challenges to descriptors and are therefore widely employed in some experiments [16, 20, 21, 32, 41]. To present the results intuitively, we classified several groups according to the occlusion rate, as shown in Fig. 7.

In addition, to comprehensively evaluate the performance of the proposed descriptor, we generated new scene-based datasets as described above. We built a scene group using several-level mesh decimations (1/2, 1/4, 1/8, and 1/16) for scenes in the Bologna dataset to separately test the robustness of the LRF to mesh decimation. The second scene group was generated by implementing different mesh decimation rates (1/2, 1/4, and 1/8) for scenes in B3R to test the performance of the descriptor. The surface of the second scene group is mixed with various rates of Gaussian noise and mesh decimation.

#### Evaluation criteria

To quantitatively evaluate the performance of the proposed LRF and descriptor, we employed *Meancos* [15, 20, 23, 32] and other methods, as well as the RPC [21–23, 32], to obtain the experimental results in this study. These methods are widely used to evaluate the performance of LRF, descriptors, and other algorithms [15, 16, 20–23, 32]. It is defined as follows:

First, we evaluate the performance of the LRF. The *Meancos* criterion is widely used to measure the mean angular error of the axes between two LRFs and is defined as follows:

17 $$MeanCos({\mathbf{F}}_{m} {, }{\mathbf{F}}_{s} ) = \frac{{\sum\limits_{i = 1,j = 1}^{n} {[CosX({\mathbf{F}}_{m,i} {, }{\mathbf{F}}_{s,j} {) + }CosZ({\mathbf{F}}_{m,i} {, }{\mathbf{F}}_{s,j} )]} }}{2n}$$

where F_*m*_ and F_*s*_ represent the corresponding LRFs of the total keypoints in the model and scene, F_*m,i*_ and F_*s,j*_ represent the LRF of the corresponding keypoints in the model and scene, *n* is the number of keypoints used for measurement, *CosX* and *CosZ* represent the cosines of the angles of the corresponding X-axis and corresponding Z-axis between F_*s,j*_ and the transformed F_*m,i*_ by the ground-truth matrix, respectively. It is not necessary to consider the Y-axis, because it is generated along the X- and Z-axes. For each experiment, we randomly selected 1000 points in the model as keypoints and generated the corresponding keypoints in the scene through a ground-truth matrix transformation. Subsequently, the LRFs of the model and scene keypoints were calculated. Finally, the *Meancos* value was computed using Eq. 17. Ideally, the axis and *Meancos* errors of a group of LRFs at the corresponding keypoints should both 1.

In addition, considering that the performance of the LRF is influenced by both the accuracy and disambiguation of each axis, we created the APX (i.e., accuracy percentage of X-axis), APZ (i.e., accuracy percentage of Z-axis), DAX (i.e., disambiguation percentage of X-axis), and DAZ (i.e., disambiguation percentage of Z-axis) to analyze the LRF performance in detail. APX, APZ, DAX, and DAZ are defined as follows:

18 $$APX({\mathbf{F}}_{m} {, }{\mathbf{F}}_{s} ) = \frac{{||CosX({\mathbf{F}}_{m,i} {, }{\mathbf{F}}_{s,j} )||}}{n \, }$$

19 $$APZ({\mathbf{F}}_{m} {, }{\mathbf{F}}_{s} ) = \frac{{||CosZ({\mathbf{F}}_{m,i} {, }{\mathbf{F}}_{s,j} )||}}{n}$$

20 $$DAX({\mathbf{F}}_{m} {, }{\mathbf{F}}_{s} ) = 1 - \frac{{N_{amb}^{X} }}{n \, }$$

21 $$DAZ({\mathbf{F}}_{m} {, }{\mathbf{F}}_{s} ) = 1 - \frac{{N_{amb}^{Z} }}{n \, }$$

where $N_{amb}^{X}$ and $N_{amb}^{Z}$ represent the number of sign ambiguations on the X- and Z-axes, respectively, in all LRFs. The higher the values of APX, APZ, DAX, and DAZ, the better the disambiguation and accuracy.

The RPC is calculated as follows: Given the model, scene, and corresponding ground-truth matrix from the model to scene, each model keypoint feature was matched with all scene keypoint features, and the closest and second-closest corresponding features were determined. If the ratio of the closest feature distance to the second-closest feature distance is less than a threshold δ, the model keypoint feature and scene keypoint feature are regarded as pairs of matches. Then, a pair of matches is regarded as a correct match if the spatial position error between the corresponding keypoints is sufficiently small (in this study, we set the error threshold to half of the value of the descriptor support radius *r*). Otherwise, the match was judged false. A curve can be calculated by changing the ratio threshold δ. Specifically, the Recall and Precision are defined as

22 $${\text{Recall = }}\frac{{\text{The number of true point matches}}}{{\text{Number of total points}}}$$

23 $${\text{1 - precision = }}\frac{{\text{The number of false point matches}}}{{\text{Number of total point matches}}}$$

If the descriptor ideally achieves both precision and recall, the RPC curve appears in the upper-left corner of the chart. To reduce the impact of keypoint sampling errors on the test results, each test experiment on the LRF and descriptors was performed five times, and the average of the results was considered the final result.

#### Parameter setting

The proposed LRF and descriptor were tested using five current LRF construction methods and eight descriptors to conduct comparative experiments. Specifically, the compared LRF methods include those of Tombari et al. [37], Mian et al. [26], Guo et al. [9], Yang et al. [13], and Ao et al. [27]. The compared descriptors included SI, FPFH, USC, SHOT, RoPS, TOLDI, SGC, and LoVS. The parameter settings of the compared descriptors are listed in Table 1, and the support radius value of the LRF is the same as that of the corresponding descriptor. Note that the SI, FPFH, USC, SHOT, and RoPS descriptor codes were obtained from the Point Cloud Library [43], the TOLDI descriptor code was acquired from Yang, and the SGC and LoVS descriptors were obtained from published papers.

**Table 1:** Parameter settings for 8 feature descriptors

	Support radius (*mr*)	Dimensionality	Length (mm)
SI	15	15 × 15	225
FPFH	15	15 × 15	225
3DSC	15	15 × 11 × 12	1980
SHOT	15	8 × 2 × 2 × 10	320
RoPS	15	3 × 3 × 3 × 5	135
TOLDI	15	3 × 20 × 20	1200
SGC	15	8 × 8 × 8 × 2	1024
LoVS	15	9 × 9 × 9	729
Proposed	15	9 × 9 × 9	729

### Proposed LRF’s performance evaluation result of and discussion

**Fig. 8:**
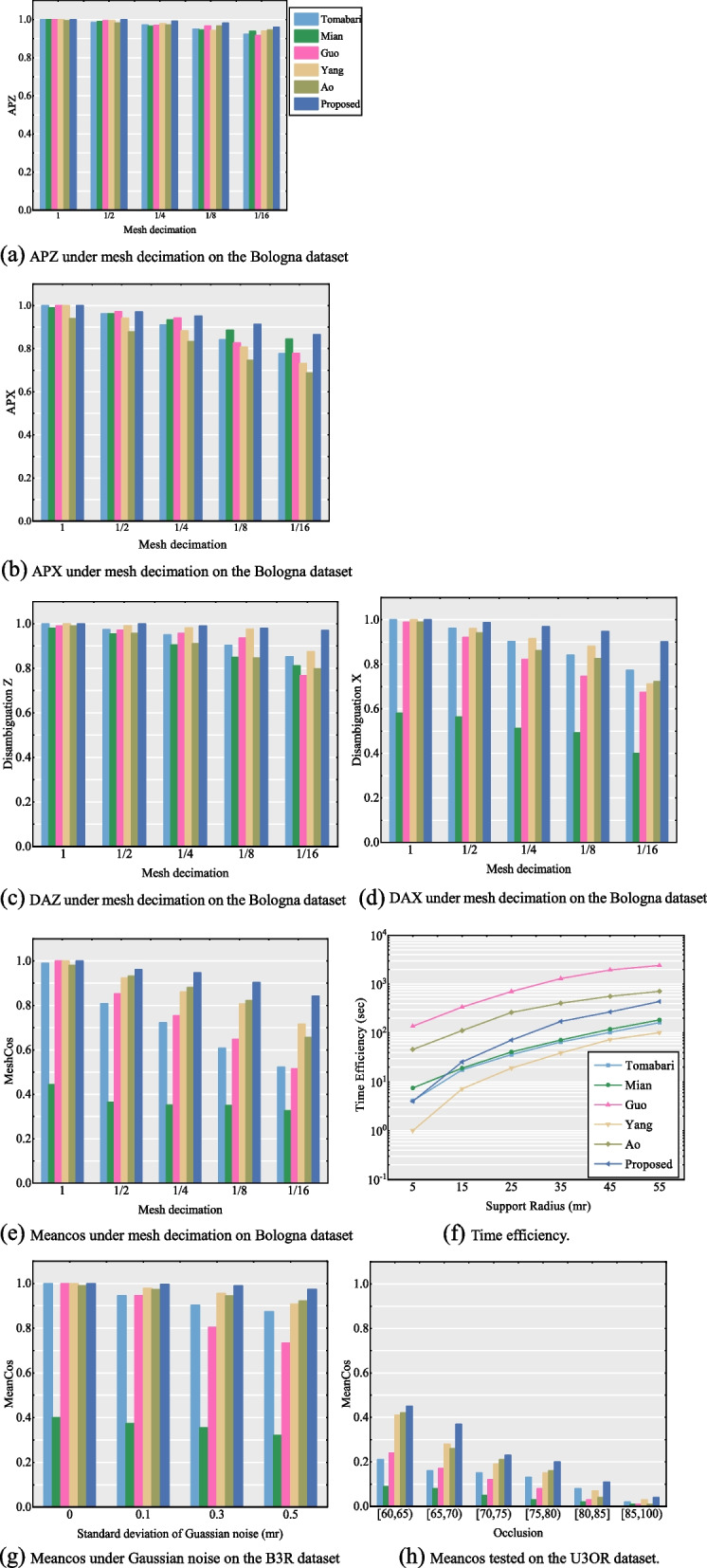
The repeatability and time efficiency performance of six LRF construction methods tested on three datasets. (**a**) APZ under mesh decimation on the Bologna dataset; (**b**) APX under mesh decimation on the Bologna dataset; (**c**) DAZ under mesh decimation on the Bologna dataset; (**d**) DAX under mesh decimation on the Bologna dataset; (**e**) Meancos under mesh decimation on Bologna dataset; (**f**) Time efficiency; (**g**) Meancos under Gaussian noise on the B3R dataset; (**h**) Meancos tested on the U3OR dataset

The performance results of the six LRF construction methods experimentally tested on the three datasets are shown in Fig. 8. In the experiments, different levels of Gaussian noise, mesh decimation, and occlusion were selected as variables. We used the Bologna dataset to test the performance of the LRF methods against different mesh decimations and time efficiencies, the U3OR dataset to test the performance against occlusion, and the B3R dataset to test the robustness of the LRF to Gaussian noise. Considering that most LRF methods are relatively robust to noise and sensitive to mesh decimation, we focused on testing the accuracy and ambiguity of each axis of the LRFs under different levels of mesh decimation. To present the results of the robustness to occlusion, we classified each model in scenes by the occlusion rate given in the initial configuration.

#### Repeatability performance

As shown in Fig. 8, compared with other current LRF construction methods, the proposed LRF method maintained an excellent performance in some experiments. Specifically, Ao’s method introduces the Gaussian standard distribution function of the neighboring points of the keypoint as the X-axis determination weight of the LRF. Although the performance was improved by employing a Gaussian function to construct the LRF, this method was relatively sensitive to mesh decimation. Compared to other methods, the performance of the method proposed by Mian et al. is not ideal because it only solves the ambiguity of the Z-axis sign of the LRF and does not address the ambiguity of the X-axis. Therefore, the method of Mian et al. performed poorly in the X-axis ambiguity test, and its overall performance was greatly limited.

The three methods proposed by Tombari et al., Guo et al., and Yang et al. considered addressing sign ambiguity for each coordinate axis using different principles; thus, the overall performance was greatly improved. The method proposed by Tombari et al. constructed a spatial scatter matrix and added a distance weight to improve its accuracy and stability. The Z- and X-axes of the LRF take the eigenvectors of the covariance matrix. Thus, the calculation of the algorithm is relatively small; yet it is quite sensitive to changing surface resolution. The method proposed by Guo et al. constructs triangular grids based on neighboring points and adds a weight related to the area of ​​the grid to improve the robustness of the surface resolution; however, this method makes the LRF relatively sensitive to noise. The method proposed by Yang et al. selects smaller subspace neighboring points of the keypoint as the input of the covariance matrix, which makes the method more stable at the boundary of the surface, as shown in Fig. 8(h).

In the experiment using the U3OR dataset, the models in the scene exhibit varying degrees of occlusion and overlap. TOLDI exhibits better stability owing to the selection of a smaller subspace. Benefitting from the appropriate weight selected, the proposed method exhibited excellent performance compared to the other methods.

Compared with the above methods, the LRF proposed in this study performed better in terms of stability and repeatability in the experiment. We applied weights to the covariance matrix and conducted effective disambiguation, which successfully improved the descriptiveness and distinguishability of each axis of the LRF. For occlusion and clutter in scenes, we consider that choosing a smaller neighborhood space improves the stability of the LRF to the local surface boundary, but it also affects the stability of the LRF [32]. To ensure the accuracy of the descriptor, we selected the original neighborhood space for feature extraction.

#### Generalization performance

**Fig. 9:**
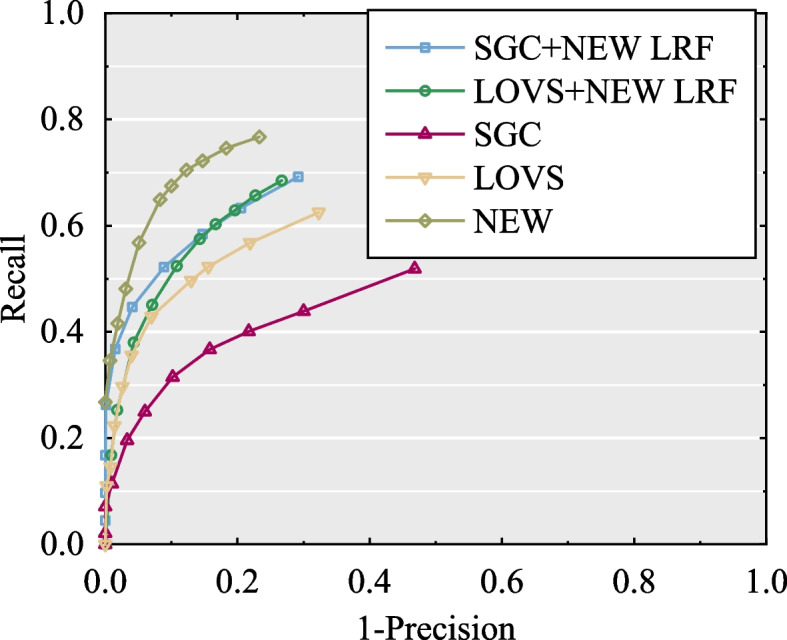
The generalization performance test of proposed LRF

To further evaluate the generalization ability of the proposed LRF, we replaced the corresponding LRF in two LRF-based descriptors, that is, the SGC and LoVS descriptors, with our LRF. This experiment was implemented using scenes from the Bologna dataset with 1/4 mesh decimation and 0.3 *mr* Gaussian noise to test the performance. The results of the original and LRF-replaced descriptors using the RPC are shown in Fig. 9.

It is evident that these two LRF-replaced descriptors perform better than the original descriptors. Therefore, the generalization ability of the proposed LRF is better, and the performance of the descriptors can be improved by employing the proposed LRF.

#### Time efficiency

We chose the Bologna dataset to test the time consumptions of the LRF construction methods. During each experiment, 1000 keypoints were randomly selected to calculate and collect the time costs of the LRF generation with several support radii. The larger the neighborhood radius, the more abundant the number of points in the neighboring space, thereby raising the calculation load involved for the covariance matrix.

As shown in Fig. 8(f), the LRF proposed by Yang et al. is the most efficient method because it selects the subspace neighboring points to generate the covariance matrix and reduces the dimensions of the covariance matrix; therefore, its calculation efficiency is faster. The LRF proposed by Tombari et al. [37] and Mian et al. [26] only needed to generate a covariance analysis to determine the LRF; therefore, the calculation efficiency was relatively high. The computational efficiency of the LRF proposed by Guo et al. is much lower than that of others because this method needs to construct a triangular mesh and perform a covariance analysis on each triangle; thus, the computation is larger than that of other methods. The LRF proposed by Ao et al. must calculate the mean of the neighboring keypoint, Gaussian function, and covariance matrix, which raises the computational load. The time efficiency of the LRF proposed in this study was slightly lower than those of Tombari et al. and Mian et al. and better than those of Guo et al. [9] and Ao et al. [27]

### Performance evaluation of proposed descriptor’s performance evaluation result of and discussion

We tested nine descriptors, including the descriptor in this study, on the Bologna and B3R datasets, under varying mesh decimation, different levels of Gaussian noise, and the situation using both mesh decimation and Gaussian noise. The experimental results of the descriptors are shown using the RPC.

#### Performance on the Bologna dataset

**Fig. 10:**
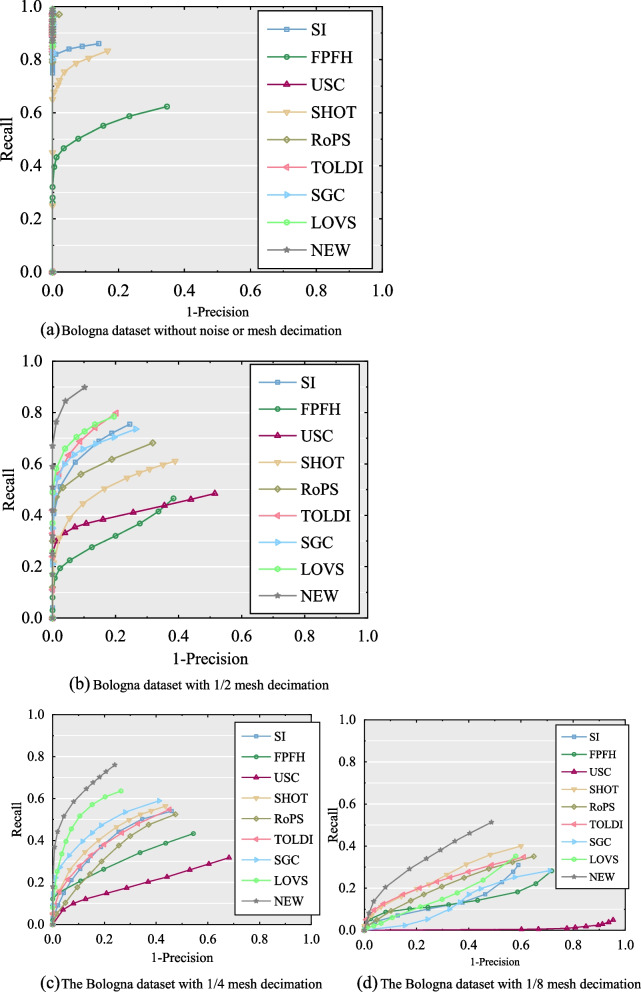
The experiment results of the performance of nine feature descriptors tested on Bologna datasets. (**a**) Bologna dataset without noise or mesh decimation; (**b**) Bologna dataset with 1/2 mesh decimation; (**c**) The Bologna dataset with 1/4 mesh decimation; (**d**) The Bologna dataset with 1/8 mesh decimation

The experimental results for the performance of the nine descriptors exhibited by RPC are shown in Fig. 10. With respect to the robustness of mesh decimation, the proposed descriptor performed better on the Bologna dataset with several different levels of decimation compared with other descriptors. The performance of the USC descriptor decreases rapidly with a gradual increase in decimation. It can be inferred that the USC descriptor extracts features depending on the number of points in each grid and consequently presents sensitivity to mesh decimation. The LoVS and SGC descriptors performed well in 1/2 and 1/4 mesh decimations, whereas they were inferior to the 1/8 mesh decimation and other higher-level decimations. In general, the performance of the SHOT descriptor was stable under varying mesh decimations.

#### Performance on the B3R dataset

Regarding the robustness to Gaussian noise, the USC descriptor performed excellently to higher-level noise compared to the others, whereas the FPFH, SHOT, and RoPS descriptors were sensitive to Gaussian noise. Specifically, it can be seen that LoVS, SGC, and our descriptors perform better at different levels of noise and follow USC. This is because these express features use point information in each voxel, and the output feature is not affected if noise and true points are dropped in the same voxel [21]. These results demonstrate that the methods of cubic splitting of neighboring spaces are significantly robust to Gaussian noise. Moreover, the SI descriptor was relatively stable with respect to different types of noise.

**Fig. 11:**
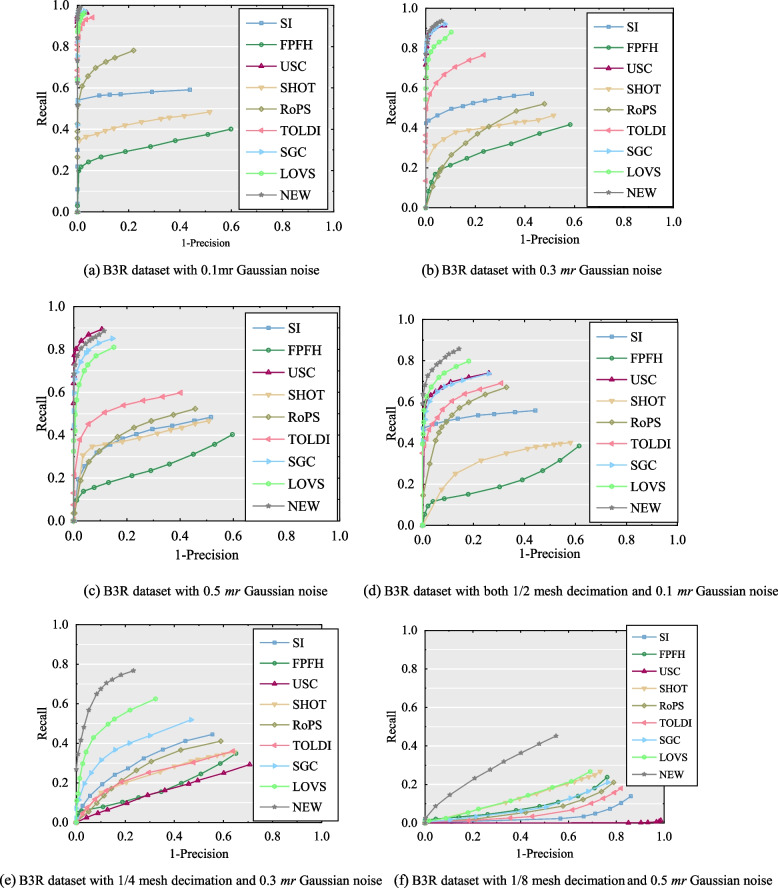
The experiment results of the performance of nine feature descriptors tested on the B3R datasets. (**a**) B3R dataset with 0.1mr Gaussian noise; (**b**) B3R dataset with 0.3 *mr* Gaussian noise; (**c**) B3R dataset with 0.5 *mr* Gaussian noise; (**d**) B3R dataset with both 1/2 mesh decimation and 0.1 *mr* Gaussian noise; (**e**) B3R dataset with 1/4 mesh decimation and 0.3 *mr* Gaussian noise; (**f**) B3R dataset with 1/8 mesh decimation and 0.5 *mr* Gaussian noise

Under the mixed interference of noise and decreased surface resolution, as shown in Fig. 11, the performance of all descriptors is greatly affected. Our descriptor achieved excellent performance compared to the others, followed by the LoVS descriptor. Although it is evident that all descriptors perform inferiorly to the 1/8 mesh decimation and 0.5 Gaussian noise, the proposed descriptor is relatively better. The excellent performance of the descriptor proposed in this study relies on its high stability and distinguishing LRF. However, it improves the stability of the descriptor by homogenizing the spatial voxel information.

#### Performance on the U3OR dataset

**Fig. 12:**
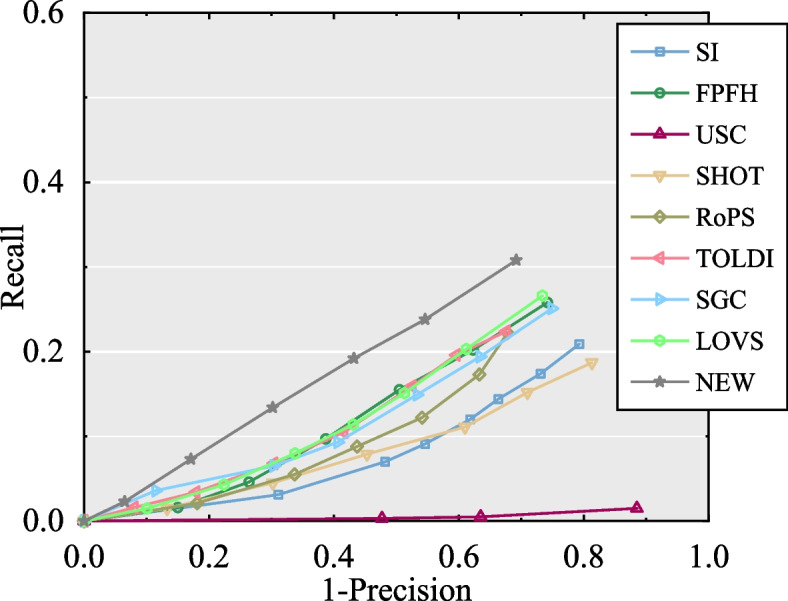
The experiment results of the performance of nine feature descriptors tested on U3OR datasets

The experimental results tested on the U3OR dataset are shown in Fig. 12. As shown in Fig. 7(c), the scenes in the U3OR dataset have varying rates of occlusion, clutter, overlap, and mesh decimation. Therefore, the robustness and descriptiveness of descriptors are rigorous challenges. Although all nine descriptors performed poorly on the U3OR dataset, the proposed descriptor achieved the best performance in complicated situations. In particular, the LoVS and SGC descriptors performed better, proving the feasibility of the spatial cubic voxel strategy for feature descriptors. Moreover, the RoPS, TOLDI, SGC, and FPFH descriptors performed well, whereas USC performed poorly.

#### Time efficiency

**Fig. 13:**
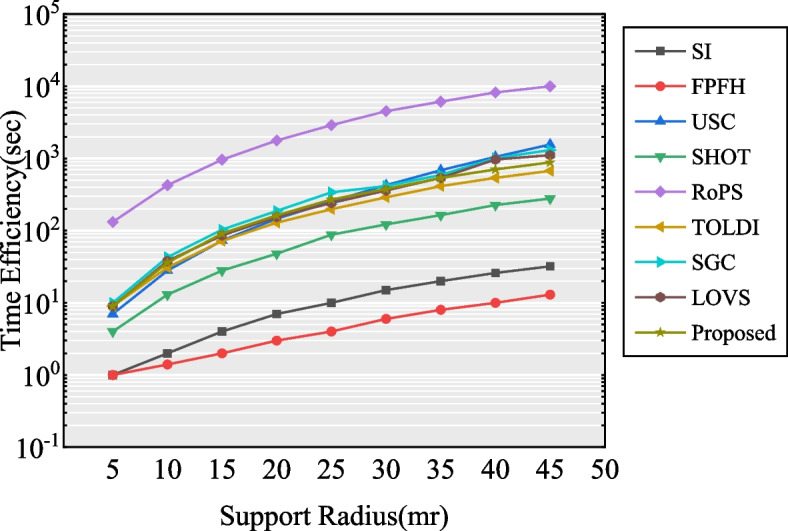
The experiment results of the time efficiency of nine feature descriptors tested on B3R datasets

The time efficiencies of nine descriptors were tested using the B3R dataset. Similar to testing for the time efficiency of the LRF, we randomly selected 1000 keypoints from each model in the B3R dataset and collected the time costs of the descriptor calculations that were implemented on these keypoints with varying support radii *r*. Note that the time efficiency is determined by the number of points in the radius of the neighboring space of the keypoints. Thus, the time efficiency can be tested by changing the value of the support radius. The experimental results are presented in Fig. 13.

The results shown in Fig. 13 conclusively indicate that the FPFH and SI descriptors exhibit excellent time efficiency, followed by the SHOT descriptor, whereas the RoPS descriptor is the most time-consuming method owing to the time cost of calculation in the LRF construction. Moreover, the time efficiencies of TOLDI, SGC, LoVS, and our descriptor are similar, and all rank fourth. Although the proposed descriptor is slightly inferior in terms of the time efficiency of SI, FPFH, and SHOT, it achieves a better performance in terms of descriptiveness and stability compared with the other descriptors.

### Proposed descriptor’s performance evaluation result of and discussion

**Fig. 14:**
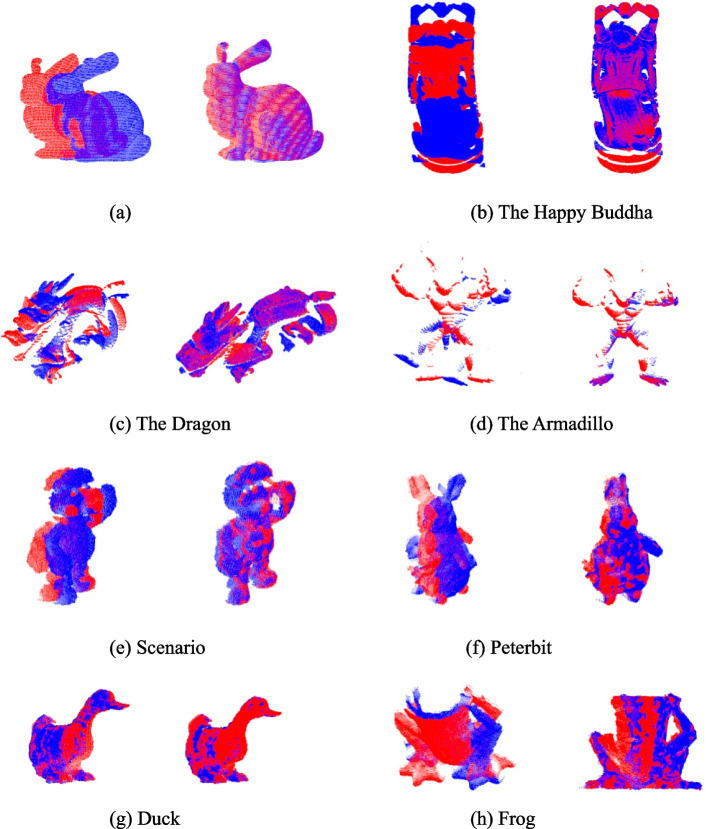
Results of 8 pairs of point clouds with various resolutions using the proposed descriptor. The left view of the red point clouds and the blue point clouds represent original and target, respectively. The right view presents the registration result of the two above point clouds using the proposed descriptor. (**a**) The Rabbit; (**b**) The Happy Buddha; (**c**) The Dragon; (**d**) The Armadillo; (**e**) Scenario; (**f**) Peterbit; (**g**) Duck; (**h**) Frog

3D point cloud registration is one of the most important aspects of 3D vision applications, and feature matching is a basic method for registration. Eight pairs of scanned point clouds are used to test the registration application using the proposed descriptor. These point clouds include ‘Bunny’, “Happy Buddha”, ‘Dragon’ and ‘Armadillo’ in Stanford Repository, and ‘Mario’, ‘PeterRabbit’, ‘Duck’ and ‘Frog’ in the Kinect dataset [23, 37, 38]. The detailed experiment is as follows. First, we selected 1000 points on the model and certain points on the scene as keypoints and used the proposed descriptor to extract the features. Subsequently, for each keypoint feature vector of the model, a correspondence was established between the feature vector of the model and the scene using the RANSAC method. The registration results are presented in Fig. 14 and Table 2. The original and target point clouds are set to red and blue, respectively. The experimental results reveal that the proposed descriptor can accurately extract features and perform matching.

**Table 2:** Registration accuracy results of descriptors experimented on two datasets

Descriptor	Stanford repository dataset	Kinect dataset
SI	31.6	0.01
FPFH	33.4	0.02
USC	49.2	0.02
SHOT	48.3	0.11
RoPS	60.7	0.17
TOLDI	67.7	0.23
SGC	73.4	0.26
LoVS	78.2	0.28
Proposed	83.4	0.30

## Conclusions

In this study, we propose a new description method that contains an LRF and a feature descriptor. The advantages of the proposed method include high descriptiveness and robustness to mesh decimation, noise, occlusion, and clutter.

The proposed LRF construction is based on the Z-axis, which is determined by the weighted covariance matrix, and the X-axis, which uses weighted projection vectors. To achieve excellent performance, we tested and analyzed the weights implemented in the LRF construction. Compared to other current methods, our LRF performed best on the three datasets with several levels of mesh decimation, noise, occlusion, and clutter. Moreover, the proposed LRF is suitable for other descriptors. On the other hand, the descriptor relies on the LRF to extract features by splitting the neighboring space into voxels and encoding the labels of these voxels. Owing to the robustness and high descriptiveness of the proposed LRF, the geometrical and spatial distribution information of the local surface encoded in the descriptor exhibited superior performance. Experiments designed to evaluate the performance show that the proposed descriptor outperforms eight current descriptors with high descriptiveness and strong robustness to noise, mesh decimation, etc. Finally, our descriptor performs well in 3D registration applications, further verifying the superiority of our descriptor.

## Data Availability

All data generated or analyzed during this study are included in this published article.

## Abbreviations

LRF: Local reference frame
SI: Spin image
3DSC: 3D shape context
FPFH: Fast point feature histograms
PFH: Point feature histograms
SPFH: Simplified point feature histograms
TOLDI: Triple orthogonal local depth images
LoVS: Local voxelized structure
LSP: Local surface patch
ISS: Intrinsic shape signatures
USC: Unique shape context
SHOT: Signature of histograms of orientations
RoPS: Rotational projection statistics
TriSI: Tri-spin-image
SGC: Signature of geometric centroids
SDASS: Statistic of deviation angles on subdivided space
U3OR: UWA 3D object recognition
RPC: Recall vs 1-precision curve
